# Mushroom Intoxication in Türkiye: A Nationwide Cohort Study Based on Demographic Trends, Seasonal Variations, and the Impact of Climate Change on Incidence

**DOI:** 10.5152/tjg.2024.24368

**Published:** 2025-01-01

**Authors:** Dilara Turan Gökçe, Derya Arı, Naim Ata, Hale Gökcan, Ramazan İdilman, Mustafa Mahir Ülgü, Murat Harputluoglu, Mesut Akarsu, Zeki Karasu, Mustafa Okan Ayvalı, Şuayip Birinci, Meral Akdoğan Kayhan

**Affiliations:** 1 Department of Gastroenterology, Ankara Bilkent City Hospital, Ankara, Türkiye; 2 General Directorate of Information Systems, Ministry of Health, Ankara, Türkiye; 3 Department of Gastroenterology, Ankara University School of Medicine, Ankara, Türkiye; 4 Department of Gastroenterology, Inonu University Medical Faculty of Liver Transplant Institute, Malatya, Türkiye; 5 Department of Gastroenterology, Dokuz Eylül University Hospital, İzmir, Türkiye; 6 Department of Gastroenterology, Ege University School of Medicine, İzmir, Türkiye; 7 Deputy Minister of Health, Ministry of Health, Ankara, Türkiye

**Keywords:** Climate change, mushroom intoxication, morbidity, mortality

## Abstract

**Background/Aims:**

Mushroom intoxication poses a considerable public health risk due to its potential for severe toxicity and fatality. This study aims to investigate demographic trends, diagnostic locations, and mortality rates of patients with mushroom intoxication.

**Materials and Methods:**

This retrospective cohort study utilized data from the National Electronic Database of the Turkish Ministry of Health. The study focused on patients without chronic liver disease or prior liver transplantation presenting with mushroom intoxication between 2018 and 2023. Demographic information, diagnostic locations, and mortality rates were analyzed, considering a six-year period to ensure even seasonal distribution.

**Results:**

Among 30 459 individuals admitted with mushroom intoxication, 44.75% were male, with a mean age of 45.84 years. The Black Sea, Marmara, and Central Anatolia regions had the highest number of cases, with specific cities like Tokat, Bolu, Yozgat, and Kastamonu having the highest rates per 100,000 population in 2022. Mushroom intoxication predominantly occurred in May, June, October, and November. Hospitalization occurred in 8.9% of cases, with a 6.6% mortality rate within 90 days and 1.3% progressing to liver transplantation. Notably, mushroom intoxication cases increased by 130% in the first half of 2023, particularly in May and June, correlating with increased rainfall.

**Conclusion:**

Mushroom intoxication is a serious public health issue, with morbidity and mortality influenced by climate factors. The study highlights a significant increase in cases in the first half of 2023, potentially linked to heightened rainfall and climate change.

**Main Points**

- Mushroom intoxication poses a considerable public health risk due to its potential for severe toxicity and fatality. This study sheds light on the influence of changing climate and precipitation patterns on the escalating global impact of mushroom intoxication.
- Mushroom biology, intricately linked to climate factors, plays a pivotal role in the seasonality of mushroom intoxication.
- Raising awareness about mushroom intoxication is imperative for both society and professional organizations to mitigate its consequences effectively.

## Introduction

The issue of mushroom toxicity stands as a significant public health concern, carrying the potential for severe intoxication and fatal outcomes. Existing studies have underscored that the majority of mushroom intoxications arise from unidentified mushroom species, with amatoxin-containing mushrooms, notably those belonging to the Amanita genus, being responsible for a considerable proportion of fatal cases.^1^ It is essential to emphasize, however, that not all mushrooms exhibit toxic properties; many are not only safe for consumption but also possess medicinal benefits.^2^

The ongoing identification and classification of toxic mushrooms remain critical, given the continuous discovery of new toxic species.^3^ To comprehend the toxic effects and mechanisms of bioactive substances in mushrooms is paramount for the prevention and treatment of mushroom intoxication.^4^ Among the hazardous mushroom species, Amanita phalloides, recognized as the Death Cap mushroom, stands out as one of the world’s most toxic and potentially lethal varieties. Its highly toxic amatoxin content, if ingested, can lead to severe liver and kidney damage.^5^ In the context of Türkiye, Amanita phalloides species is identified as the primary culprit behind mushroom intoxication, given its prevalence and the significant gravimetric potency of its toxins, resulting in a higher incidence of lethal mushroom intoxication compared to other species.^6^

**Figure 1.**
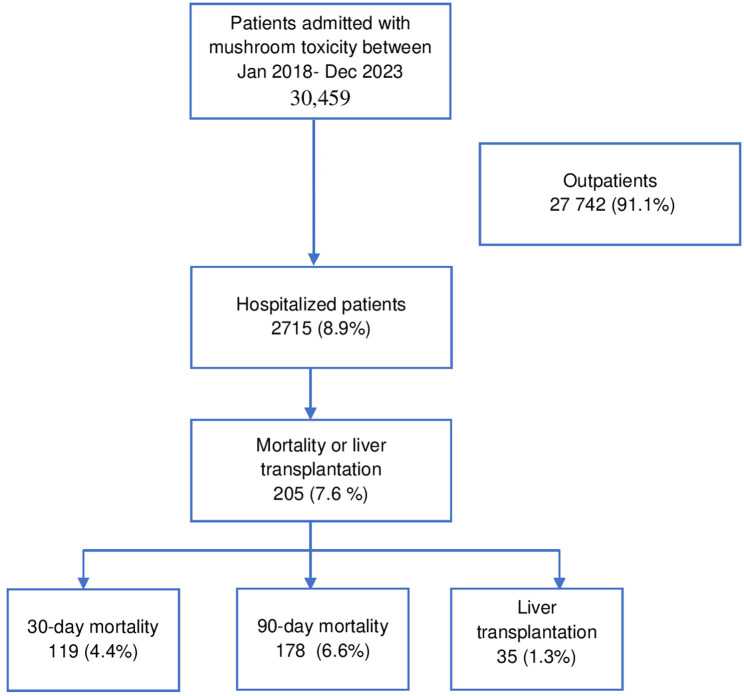
Flow chart: The clinical course of patients who were admitted to the hospital with mushroom intoxication.

Mushroom toxicity, a severe medical condition with the potential to cause liver damage, sometimes requiring liver transplantation (LT), necessitates a profound understanding of patient characteristics and outcomes.^7^ Epidemiological data play a pivotal role in comprehending the incidence and distribution of mushroom intoxications, thus informing strategic public health interventions.^8,9^ As there is an evident need for enhanced insights into mushroom toxicity and the development of effective preventive strategies, this study endeavors to bridge the existing knowledge gap. It aims to investigate demographic characteristics, diagnostic locations, and mortality rates, with a primary focus on unveiling the frequency and geographic distribution of mushroom-related toxicity in Türkiye. Additionally, the study delves into the potential influence of the climate crisis on mushroom intoxication within the Turkish population (Figure 1).

## Material and Methods

This multi-center retrospective cohort study utilized data from the National Electronic Database of the Turkish Ministry of Health to investigate cases of mushroom intoxication (T62.0) between January 1, 2018, and December 31, 2023. Patients with chronic liver disease or a history of liver transplantation were excluded from the analysis. The study focused on patients’ characteristics, diagnostic locations, and assessed 30-day and 90-day mortalities. Additionally, the study explored whether patients underwent liver transplantation (LT) within 90 days, and the medical treatments and supportive therapies received by these patients were evaluated.

The Ministry of Environment, Urbanization, and Climate Change of the Republic of Türkiye has examined the annual precipitation analysis reports published by the General Directorate of Meteorology. The existing precipitation data in our country have been reviewed based on these reports. The research protocol obtained approval from the Ministry of Health of Türkiye.(approval no: 95741342-020; date: 27.11.2019). Since the study is a national data and retrospective study, written informed consent was not obtained from the patients.

### Geographical Considerations

Türkiye, with its diverse geography and culture, is divided into seven regions: Marmara, Aegean, Mediterranean, Central Anatolia, Black Sea, Eastern Anatolia, and Southeastern Anatolia. The number of mushroom intoxication cases was analyzed in correlation with these regions, offering insights into potential regional variations.

### Nationwide Population-Based Study

This study, conducted as a nationwide population-based retrospective cohort study, adhered to rigorous ethical standards. The research protocol obtained approval from the Ministry of Health of Türkiye under the identification number 95741342-020 (approval date: 27.11.2019). The design and execution of the study followed the principles outlined in the Declaration of Helsinki, ensuring ethical standards and participant safety. Since the study is a national data and retrospective study, written informed consent was not obtained from the patients.

### Statistical Analysis

The statistical analyses were conducted using Jamovi version 2.3.19.0 (The Jamovi Project; Sydney, Australia). Continuous variables were expressed as median and range, while categorical variables were summarized with counts and percentages. For between-group comparisons, the chi-square test (or Fisher exact test, when appropriate) was used for categorical variables and the Mann-Whitney U test for continuous variables. *P* values were reported from nonparametric t-tests or the chi-square test. Statistical significance was set at *P* < .05.

## Results

### Demographic Characteristics

A total of 30 459 individuals were affected by mushroom intoxication. The gender distribution revealed that 44.75% were male, with a mean age of 45.8 ± 20.3 years. Pediatric cases, comprising individuals 18 years or younger, accounted for 10.97% of the total. The majority (97.9%) of cases involved Turkish nationals, while 2.1% were foreign nationals, totaling 576 individuals.

### Annual Incidence Rates

**Figure 2.**
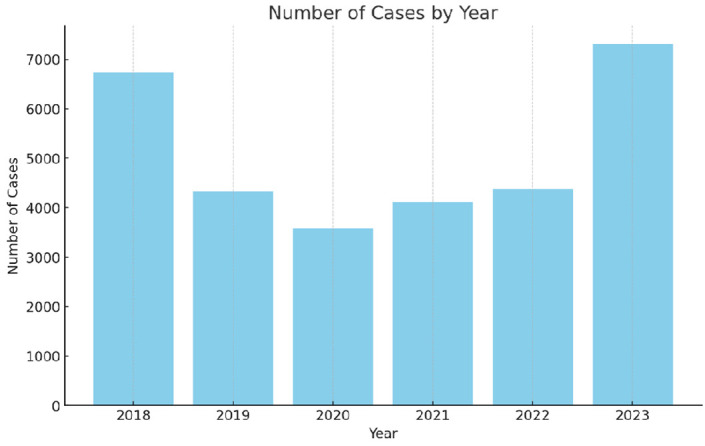
Number of cases by year.

The annual case incidence demonstrated a fluctuating pattern over the 6-year period (Figure 2). There were 6741 cases in 2018, 4339 cases in 2019, 3573 cases in 2020, 4115 cases in 2021, 4378 cases in 2022, and 7313 cases in 2023.

### Hospitalization and Liver Transplantation

Of the total cases, 8.9% (n: 2,715) required hospitalization. Among hospitalized patients, 35 individuals underwent liver transplantation due to mushroom intoxication-related acute liver failure, including 4 pediatric cases (ages: 6, 7, 9, 12). The ages of the patients who underwent liver transplantation ranged from 6 to 74 years. Eight patients were observed to have a fatal outcome within the first 90 days following the liver transplant.

The patients’ ages ranged from 4 to 74 years, with a mean age of approximately 45 years. The 25th percentile was 29.5 years, the 50th percentile (median) was 53 years, and the 75th percentile was 60.5 years. 51.4% of the patients were female, while 48.6% were male. The time between admission and transplantation varied, with a mean of approximately 2.6 days. The majority of patients had a transplantation within 0 to 2 days after admission, with some cases extending up to 29 days.

### Mortality Rates

The 30-day and 90-day mortality rates among hospitalized patients were 4.4% (n:119) and 6.6% (n:178), respectively. Patients with a fatal outcome were significantly older than survivors (63.3 ± 18.75 vs. 45.7 ± 20.8, *P* = .001) (Figure 1). A total of 205 patients (7.6% of hospitalized cases) had fatal outcomes or progressed to liver transplantation.

### Geographical Distribution

**Figure 3.**
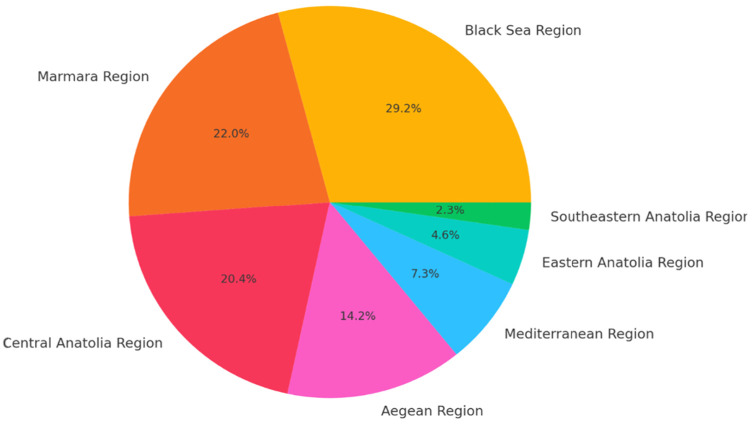
Percentage distribution of mushroom intoxication cases by region.

**Figure 4.**
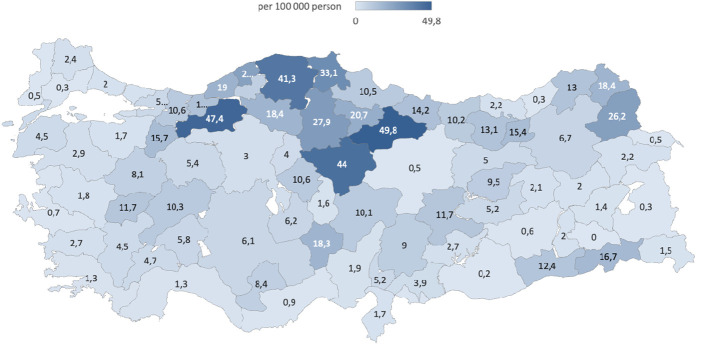
Distribution of cases in 2022, calculated per 100 000 people for each city.

Among 30 459 individuals in the six-year period, cases were distributed across diverse age groups and regions. Geographical variations were observed, with the highest case numbers in the Black Sea (29.2%), Marmara (22%), and Central Anatolia (20.4%) regions (Figure 3). In 2022, the cities with the highest rates per 100,000 population were Tokat (49.8%), Bolu (47.4%), Yozgat (44%), and Kastamonu (41.3%) (Figure 4). 

### Seasonal Trends

**Figure 5.**
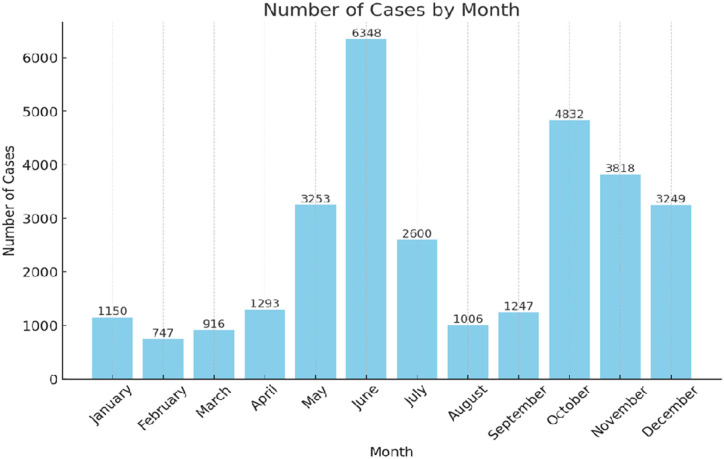
Monthly distribution of mushroom intoxication in Türkiye. The bar represents the sum of patients diagnosed during the same year throughout the study period (2018-2023).

**Supplementary Figure 1:**
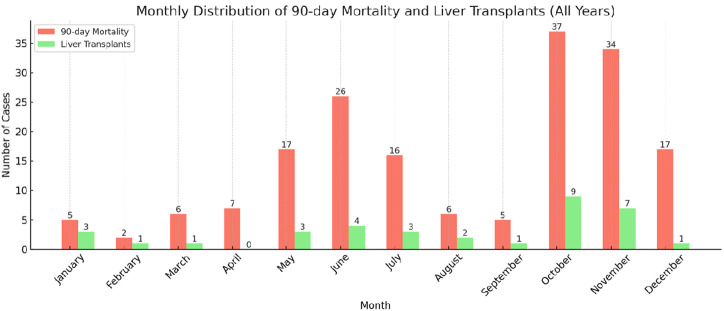
90-day Mortality and Liver Transplants (All Years) 90-day Mortality: The number of patients who died within 90 days after hospitalization each month across all years. Liver Transplants: The number of patients who underwent liver transplantation each month across all years.

Mushroom intoxication was most prevalent in June, October, and November during the study period (Figure 5). An analysis of liver transplantation and mortality by month shows that the months with the highest number of cases, May, June, October, and November, are also the months with the highest rates of transplantation and mortality (Supplementary Figure 1).

### Supportive Treatments

A total of 30 466 patients were analyzed, with the following interventions applied: gastric lavage was performed on 4,191 patients (13.76%), hemodialysis was administered to 254 patients (0.83%), and plasma exchange was utilized in 125 patients (0.41%).

A significant association was observed between hemodialysis and both 90-day mortality (*P* < .01) and liver transplantation (*P* < .01). Similarly, plasma exchange was significantly associated with 90-day mortality (*P* < .05) and liver transplantation (*P* < .01). In contrast, no statistically significant association was found between gastric lavage and either 90-day mortality or liver transplantation, with *P*-values of .51 and 1.0, respectively.

## Discussion

Mushroom intoxication is a severe public health issue influenced by climate factors. The study highlights a substantial increase in cases in 2023, especially in the first half, likely linked to heightened rainfall and possibly changing environmental landscapes. Throughout the 72-month observational period, a total of 30 459 individuals were diagnosed with mushroom intoxication, emphasizing the substantial public health threat posed by this issue. The incidence rate per 100 000 population in 2022 stood at 5.14, and for 2023, this number was observed to be 8.5 per 100 000 people, with annual case incidences exhibiting variations: 6743 cases in 2018, 4340 in 2019, 3578 in 2020, 4116 in 2021, 4386 in 2022, and 7313 in 2023. The global prevalence of mushroom intoxication diverges, exemplified by The National Poison Data System in America, reporting 7428 cases annually over an 18-year analysis.^10^ Similarly, a 20-year retrospective study in Italy detailed 12813 suspected mushroom intoxication cases, where 62.2% displayed early symptoms, 25.5% experienced delayed gastrointestinal symptoms, and 637 cases were identified as amatoxin intoxication, leading to 19 liver failure cases and 40 fatalities.^11^

In a 10-year study on pediatric cases in Türkiye, researchers identified 39 patients, 9 of whom had a fatal outcome.^12^ Mushroom intoxication is a global issue, reported in countries such as China, the USA, and Europe, with varying rates of severe intoxication and mortality. A meta-analysis of 33 studies estimated a mortality rate of up to 20%, with the necessity for liver transplantation ranging from 0.6% to 24%.^13^ However, it’s crucial to acknowledge potential variations in disease characteristics among different study groups. As expected, the study found an association between plasma exchange and hemodialysis with mortality and liver transplantation in cases of mushroom intoxication. We know that both supportive procedures are linked to multiorgan failure and disease progression.

Mushroom biology, intricately linked to climate factors, plays a pivotal role in the seasonality of mushroom intoxication. An investigation in southwestern Yukon demonstrated the predictability of epigeous mushroom production based on rainfall patterns over a 15-year period. In particular, June rainfall of 2023 year and May rainfall of the previous year were identified as predictors.^14^ In tandem with these findings, our study witnessed a 130% surge in mushroom intoxication during the initial half of 2023, notably in May and June. This aligns with previous research linking increased rainfall to elevated mushroom toxicity cases compared to the preceding year. According to data collected and analyzed by the Turkish Statistical Institute and the General Directorate of Meteorology, the highest number of cases was observed in 2018 and 2023 over the 6-year period examined. Meteorological data show that 2018 and 2023 were the years with the highest rainfall in the last 10 years. Additionally, these two years experienced above-normal precipitation.^15^ Spring rainfall in Türkiye has reached a 63-year high in 2023, with a 45% increase compared to normal levels and a 73% increase compared to the previous year.^15^ In 2018, it was observed that the total number of cases remained higher compared to the subsequent years of 2019-2022. Detailed examination of precipitation data according to the years, based on the Turkish State Meteorological Service, revealed that in 2018, the amount of precipitation increased compared to the years 2019, 2020, and 2021, surpassing the normal precipitation level considered to be above 573 mm.^16^

The rise in mushroom intoxication cases may indeed be tied to favorable environmental conditions for mushroom growth facilitated by heightened rainfall. The study emphasizes the critical role of public health authorities in raising awareness about the risks associated with consuming wild mushrooms. Education on proper identification and handling is crucial to prevent mushroom intoxication incidents.

However, it is imperative to contextualize these findings within the broader socio-environmental landscape. Notably, in February 2023, Türkiye experienced a significant earthquake that has had far-reaching consequences on societal dynamics, potentially influencing the patterns of mushroom intoxication. Although we do not have any reliable data on this issue, we can speculate that the aftermath of the earthquake may have contributed to shifts in dietary habits as individuals seek more affordable and readily available food options. In times of crisis, there is often a heightened reliance on easily accessible food sources, including foraging for wild mushrooms. This shift in behavior could be a contributing factor to the observed increase in mushroom intoxication cases in the first half of 2023, particularly in May and June. The socio-economic implications of the earthquake may have inadvertently driven individuals towards riskier dietary choices, thereby amplifying the incidence of mushroom intoxication.

Despite the study’s insights, this retrospective study has limitations, notably the inability to assess the severity of patients’ symptoms, which is crucial for understanding the disease’s impact on individuals and the healthcare system. Another important limitation is the inability to assess the cost of the disease and the socioeconomic status of individuals, which restricts the scope of the analysis in these areas.

In conclusion, mushroom intoxication, prevalent during the wet season, poses a significant public health threat due to its associated morbidity and mortality. This study sheds light on the influence of changing climate and precipitation patterns on the escalating global impact of mushroom intoxication. Raising awareness about this issue is imperative for both society and professional organizations to mitigate its consequences effectively. This study is the first in our country to provide data on the prevalence, number, and associated mortality and liver transplantation rates related to mushroom toxicity. Its significance lies in its national cohort, offering crucial insights into this area. Public health authorities should raise awareness about the risks of consuming wild mushrooms and provide education on proper identification and handling to reduce incidents of mushroom intoxication.

## Supplementary Materials

Supplementary Material

## Data Availability

The data that support the findings of this study are available on request from the corresponding author
