# Supplementary material for: Mushroom Intoxication in Türkiye: A Nationwide Cohort Study Based on Demographic Trends, Seasonal Variations, and the Impact of Climate Change on Incidence
Source: Turk J Gastroenterol. 2025 Jan 1;36(1):61–6. doi: 10.5152/tjg.2024.24368 (PMC11736803; doi:10.5152/tjg.2024.24368)
Supplement: Supplementary Material [file supplementary_material.pdf]

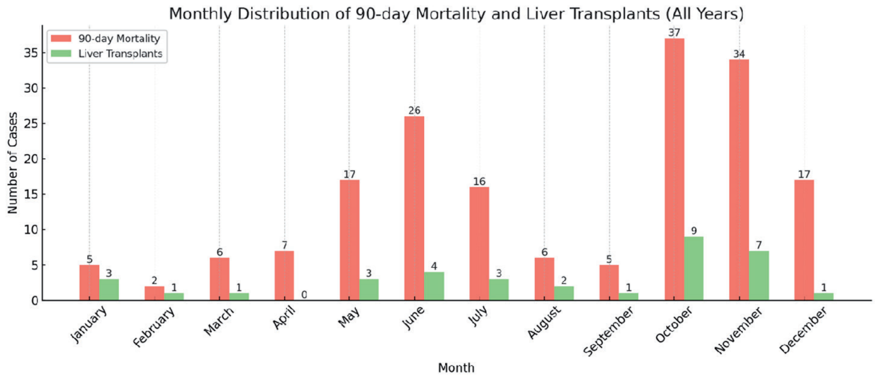

Supplementary Figure 1 90-day Mortality and Liver Transplants (All Years) 90-day Mortality: The number of patients who died within 90 days after hospitalization each month across all years. Liver Transplants: The number of patients who underwent liver transplantation each month across all years.
